# Management equity incentives and stock price crash risk: “Golden handcuffs” or “gold watch”

**DOI:** 10.1371/journal.pone.0249900

**Published:** 2021-04-26

**Authors:** Xiaohua Zhou, Jinshi Wan, Yi Yang, Xiangyu Gan

**Affiliations:** 1 School of Economics and Business Administration, Chongqing University, Chongqing, China; 2 Cardiff Business School, Cardiff University, Cardiff, United Kingdom; University of Almeria, SPAIN

## Abstract

This paper expands the previous research on management equity incentives (MEIs) and stock price crash risk by distinguishing between the "gold watch" region and the "golden handcuff" regions in MEIs. By using an estimation of the gold watch region and the golden handcuff regions based on 6,675 annual observations of China’s A-share listed companies, the stock price crash risk is found to be negatively correlated with MEIs in the golden handcuff regions (0–10%, 30%-100%) and is positively correlated with MEIs in the gold watch region (10%-30%). A further investigation of the mediating effects of peer effects on MEIs and the stock price crash risk reveals that peer effects have a partial mediation effect at the level of peer managers’ shareholding and mediate the relationship between MEIs and the stock price crash risk.

## Introduction

China’s transformation from its original planned economy system to the market economy system has been a gradual transition. China proposed a mixed-ownership reform plan in the 1990s with the aim of introducing private capital to break the complete monopoly of the state in terms of share ownership and promote the modernization of corporate governance in China. The key to the mixed-ownership reform has been the re-design of ownership structures. In the midst of the mixed-ownership reform, the internal capital structure of listed companies is constantly changing. Against this background of mixed-ownership reform for China’s listed companies, The China Securities Regulatory Commission issued “Management measures for the equity incentives of listed companies” in 2015 to encourage management to expand shareholding. Compared with that in Western countries, the research on management ownership in China is more commonly classified as an equity incentive issue. As part of the modernization of listed companies’ corporate governance in China, management ownership is following an upward trajectory. However, academia has overlooked a major theoretical and practical issue, that is, the relationship between management equity incentives and stock price crash risk. According to existing studies, the root cause of stock price crash risk lies in the fact that management conceals bad news related to its company [1–3]. Management is a major player in the release of profit forecast information, and the characteristics of the management determine the accuracy of the profit forecast information [4]. According to principal-agent theory, greater MEIs can better align the interests of managers and shareholders and alleviate the agency problem between the two parties [5, 6]. However, such an argument ignores the possibility that greater managerial autonomy leads to greater managerial opportunism. Some researchers believe that managers with larger shares have greater control over the company, which gives them more room to act in their own private interest [7, 8]. Greater MEIs mean that managers’ wealth is more sensitive to stock prices, which prompts managers to conceal bad news and mislead investors into driving up current stock prices at the expense of long-term company value. Using a dynamic rational expectations model with asymmetric information, Benmelech et al. [9] showed that while stock-based compensation causes managers to work harder, it can also tempt them to conceal bad news about future growth opportunities. Such concealment of bad news can lead to serious overvaluation and a subsequent stock price crash. This study further argues that there are differences in the impact of different levels of MEIs on stock price crash risk. MEIs both raise and lower the stock price crash risk, which can be explained by management entrenchment theory. According to this theory, executive shareholding is a means to reduce agency costs by promoting management interests in line with shareholders’ interests [10]. This occurs because managers with larger shares have a greater incentive to act in the interest of the shareholders [8, 11]. However, at a higher level of management ownership, such practices may become deeply ingrained and provide more room for self-interest, thereby increasing agency costs [7, 8, 12]. A few studies have suggested that institutional costs decrease as MEIs increase and argue that the different effects of promotion and suppression (based on interest convergence and consolidation assumptions) on agency costs provide a framework for a detailed investigation into the relationship between MEIs and stock price crash risk [13, 14].

External peer influence plays a key role in restricting management violations. Previous studies have been based on empirical investigations, and since early audit studies have focused on the level of internal corporate governance, that topic will not be covered here. In existing studies, a company is usually considered an independent unit that formulates a dividend policy to maximize the company’s interests according to its actual situation such as its growth, profitability, and capital structure. With the ever-accelerating pace of informatization and increasingly fierce market competition, companies are no longer isolated from one another, and the behaviour of peers is an important guide for company decision-making. Therefore, the research on peer effects has extended from the field of sociology to the field of economics, and research on peer effects in the discipline of corporate finance within the field of economics has been gradually on the rise. Companies are the main performers of social and economic activities, their management’s financial activities and equity allocations can be affected by relevant “peer” companies, and management’s investment and financing decisions can have a direct impact on stock price crash risk. When some companies in the same industry (especially leading companies) increase their level of debt, it can have a positive spill-over effect on the capital structure of other companies in the same industry, resulting in the companies in that industry competing to increase their level of debt and increasing the systemic risk of debt default within the industry. In this case, herd deviation exists at the industry and company levels [15]. Leary and Roberts [16] studied corporate financial decision-making and found that corporate financial decision-making can be significantly affected by peer companies and that peer effects are the largest factor affecting corporate financial decision-making. Foucault & Fresard [17] examined the roles of peer effects in the process of corporate investment decision-making, while Huang & Zeng [18] constructed a theoretical model to explain peer effects. Corporate management incentives are another important factor affecting peer effects. Patnam [19] found that there are peer effects in investment decisions and management compensation in Indian companies. Parsons et al. [20] provided further evidence to support the conclusions of Patnam [19] and attributed these peer effects to interactions among executives of listed companies. Agents have control over the operation of the company, management is confronted with enormous uncertainty risks in formulating corporate policies, and information on the corporate decision-making of peer companies has a vital impact. To enhance the competitiveness of their companies in the capital market, managers motivated to refer to the corporate decisions of other companies in the same industry, and peer effects can create norms for the behaviour of corporate managers and reduce stock price crash risk. These studies break the assumption found in previous studies that corporate decisions are independent of each other.

This study sets out to investigate the impact of MEIs on stock price crash risk. This study may contribute to the existing literature in the following ways. First, this study provides new evidence on the relationship between the MEIs of Chinese listed companies and stock price crash risk and enriches the existing literature by investigating how corporate internal governance affects stock price crash risk. We obtain unique turning points (10% and 30%) in the range of Chinese listed companies’ MEI percentages. Previous studies have shown that there is a negative relationship between MEIs and stock price crash risk. By identifying the structure of MEIs, this paper argues that this negative relationship mainly exists in "golden handcuff" regions, that is, the convergence-of-interests regions for management and shareholders. The term "golden handcuffs" refers to companies’ use of expected income from a stock option bonus to retain senior managers and other talent. As an incentive tool for senior executives, golden handcuffs aim to stimulate their motivation and share the fruits of growth with other stakeholders. And MEIs increase stock price crash risk in the "gold watch" region, that is, the management entrenchment region. The term "gold watch" refers to a situation in which management conflicts with other shareholders due to insufficient constraints and excessive motivation in the process of pursuing an incentive. In this situation, the selfishness and greed of management means that the equity incentive does not reduce agency costs but increases them. Second, this study provides the first empirical evidence for industrial peer effects on stock price crash risk through a three-step intermediary effect model, identifies the relationship between management equity incentives and stock price crash risk in the special business environment in China, and details the findings on the intermediary role of peer management equity incentives. This is crucial because each industry has its own unique characteristics, “a specific constraint relationship” [21], that is, management’s discretion in making key strategic decisions. The rest of this paper is organized as follows. The literature is reviewed, and the research hypothesis is proposed in Section 2. Details of the research design are shown in Section 3, the empirical results are discussed in Section 4, and Section 5 concludes.

## Literature and hypotheses

### Management equity incentives and stock price crash risk

A stock price crash refers to the phenomenon in which a stock price drops sharply and the stock return is extremely negative due to a sudden release of negative information or a sharp change in investor sentiment [1, 22]. Hong and Stein’s [23] theoretical model further noted that due to restrictions on short selling, bearish investors are unable to fully reflect private information in stock prices through trading, and when the market shows signs of faltering, the cumulative negative information is released all at once, resulting in a sharp drop in stock prices. Existing studies have focused on the natural characteristics of management, such as age, education, professional background and so on [24], and the literature on the self-interested motivation of management has focused on the influence of this motivation on the timing of profit forecast releases [25], their frequency [26] and their mode [27]. The existing literature has also suggested that management tends to “hold back unpleasant information” [28, 29], and company management often delays the release of bad news to protect its position and compensation [30–32], avoid taxes [22], increase the value of options in the short term [22], and create an empire [2]. However, shareholders have to pay for management fraud. For example, managers may conceal negative news to keep their jobs, which prevents shareholders from identifying operational problems in a timely manner; management secures its ability to exercise options by delaying the disclosure of bad news or fraudulently receives performance pay that should not have been paid. These directly harm the interests of shareholders. When there comes a point at which the bad news can no longer be concealed, that bad news is released all at once, exerting a massive negative influence on stock prices. Ball [33] studied the collapse of the Enron Corporation and found that the management’s desire to become public figures prompted them to exaggerate the performance of the company, and when the whitewashed image of the company was exposed, the company’s share price plummeted.

According to management entrenchment theory, if MEIs are taken as a single variable, without considering their special structure, which reduces the research significance of this topic. To improve the application value of this research, a distinction is made in terms of the percentage of equity held by managers. We divide MEIs into golden handcuff regions and a gold watch region. In general, China’s listed companies have lower levels of manager ownership. From a practical perspective, given the progress of China’s mixed-ownership reform, the MEIs of China’s listed companies have been trending upwards, from low MEI golden handcuff regions to a gold watch region, which may increase the risk of a stock price crash. Therefore, the following is hypothesized:

H1a: In golden handcuff regions, there is a negative correlation between stock price crash risk and management equity incentives.
H1b: In the gold watch region, stock price crash risk is positively correlated with management equity incentives.

### Peer effects and stock price crash risk

Peer effects were initially a hot topic in international sociological research and refer to an individual’s behaviour being affected by the behaviour of other individuals whose status is the same or similar. Winston and Zimmerman [34] claimed that peer effects exist if a person’s behaviour is affected by the behaviour of one or more other persons. With the development of econometrics, finance and other disciplines, peer effects occurring primarily at the corporate level have become a focus of research in the field of economics. The investment and financing decisions of management have a direct impact on the risk of a stock price crash, while peer effects affect managers’ financing decisions. In terms of corporate financing behaviour, Leary and Roberts [16] found that there are peer effects in the asset-liability ratio of American companies and the average asset-liability ratio of an industry, and corporate financing decisions are significantly affected by those of other companies in the same industry. Dougal et al. [20] collected data from 20 regions and 12 major industries in the United States from 1970 to 2009 as research objects and found that there were different degrees of peer effects in the same industry in the same region, in the same industry in different regions, and in different industries in the same region, and the peer effects on the investment behaviour of companies in the same industry and in the same region are the most significant. Peer effects also play a role in company management incentives. Francis [35] claimed that other companies in the same industry can effectively alleviate the agency problem, and when the quality of similar companies is higher, executives can be encouraged to work harder and improve corporate performance. Patel et al. [36] argued that managers can easily “free ride” on industry information and that this also occurs between managers. A mutual comparison of performance leads managers (especially those at an informational disadvantage) to use the decisions of peer companies as important sources of information. Managers may imitate the capital structure of other companies and free ride on other managers’ information. Due to information asymmetries, when faced with uncertainties, company managers tend to refer to the behaviour of peer companies to avoid making the worst decision. Therefore, the code of conduct brought about by peer effects also leads to certain constraints on company managers. According to the above research, another possible mechanism through which the company’s capital structure could be affected by peer companies is the capital structure and fundamental characteristics of peer companies, which contain effective information, as managers can increase the effectiveness of their capital structure decisions by referring to peer company information. Therefore, that the following is hypothesized:

H2: Peer effects mediate the influence of management equity incentives on stock price crash risk.

## Research design

### Data collection

Accounting and stock price data come from the CSMAR database. The original data set includes annual observations of companies listed on the Shanghai Stock Exchange and Shenzhen Stock Exchange between 2008 and 2017. After deleting observations of financial institutions and ST/ST* listed companies as well as observations that lack data, a final sample of 6,675 annual observations of companies within all industries was obtained. The institutional environment data, NERI, provides the registration location for the listed companies in China and comes from the "Report on China’s Marketization Index by Province (2018)". To reduce the adverse effects of extreme outliers, we winsorize all continuous variables at the 1% and 99% levels.

### Variable descriptions

#### Stock price crash risk

Following the method of Kim et al. [22], stock price crash risk is measured as both the negative return skewness coefficient (NCSKEW) and the ratio of positive and negative stock return fluctuations (DUVOL). The process for calculating NCSKEW is as follows.

First, the specific rate of return for stock i during week t, wit = ln (1+ξit), is determined, where ξit is the estimated residual term from the following model:

$$\gamma_{it}=\alpha_{i}+\beta_{1i}*\gamma_{mt}{}_{-2}+\beta_{2i}*\gamma_{mt-1}+\beta_{4i}*\gamma_{mt+1}+\beta_{5i}*\gamma_{mt+2}+\xi_{it}$$ (1)

*γ*_*it*_ is the rate of return for individual stocks in week t and the weighted average rate of return for the market in week t. Additionally, in line with the method of Dimson [37], the lead and lag terms for the market weighted average rate of return are added to the model to reduce deviations due to nonsynchronous trading. Second, the NCSKEW is calculated by using the calculated specific rate of return. The higher the value of NCSKEW is, the greater the crash risk. The calculation process for NCSKEW is as follows:

$$NCSKEW=-[n{\left(n-1\right)}^{\frac{3}{2}}\sum w_{it}^{3}]/[(n-1)(n-2){\left(\sum w_{it}^{3}\right)}^{\frac{3}{2}}]$$ (2)

where n is the number of weeks in which stock i is traded each year. The calculation process for DUVOL is as follows:

$$DUVOL=\log\frac{(n_{u}-1)\sum_{down}w_{it}^{2}}{(n_{d}-1)\sum_{up}w_{it}^{2}}$$ (3)

where nu (nd) is the number of weeks in which the weekly return of stock i is higher (lower) than the average rate of return for the year. The interpretation of DUVOL is consistent with that of the NCSKEW index; that is, the greater the value of DUVOL is, the greater the stock price crash risk.

#### MEI

Following previous studies [38, 39], the MEI in this paper is defined as the proportion of common shares owned by executive directors or senior managers, including members of the board of directors, the general manager, the deputy general manager, the chief financial director, the chief engineer, the chief economist, the chief agronomist, the secretary of the board of directors and members of the board of supervisors. A series of segments were tested, and the turning point in the MEI entrenchment region was identified to determine the range of the low MEI region (MEI_L), intermediate MEI region (MEI_M), and high MEI region (MEI_H). As with previous studies in which piecewise regressions were used to evaluate MEIs (for example, Lennox [40]; Morck et al. [41]), this study defined three ownership regions: MEI_L (the interval of low MEI percentages, which is usually considered to be a golden handcuff region), MEI_M (the interval of medium-level MEI percentages, which is usually considered to be a gold watch region) and MEI_H (the interval of high MEI percentages, which is usually regarded as a golden handcuff region). As far as we know, there are currently no studies of the gold watch MEI region in China, so trial and error methods were used in this study. Using regression model 4, the impact of gold watch regions on stock price crash risk was examined based on the entrenchment regions discussed in previous studies. For example, 21%-50% (Australia), 5%-25% (US), 12%-40% (UK), and 20%-50% (Hong Kong, China) were tested. In addition, as the MEIs in China’s stock market are lower than those in developed stock markets, a number of entrenchment regions, including 5%-25%, 10%-40% and 25%-50%, were also tested. The key purpose behind returning to the hypothetical relationship between these regions is to find further evidence to support the robustness of the main results for hypotheses 1a and 1b. These tests demonstrate that the entrenchment region that best describes the Chinese stock market sample is 10%-30%, and the turning points are significant at the 10% level at least. Although these cut-off points are different from those in previous estimates, their trend is consistent. The different turning points estimated in this study reflect the background differences in share ownership and suggest the importance of accounting for jurisdictional environment in the field of corporate governance research, which is further tested in Hypothesis 2.

#### PMEI

Furthermore, peer management equity incentives (PMEIs) are used to measure the shareholding level of peer managers, that is, the average annual MEI of the industry excluding the company’s own MEI. According to a previous literature review [36], two mechanisms affect company policies on management equity incentives: imitation based on learning through communication among executives and imitation based on observational learning. Following Ahern et al.’s [42] research design ideas for peer effects, the calculation process is as follows:

$$PMEI_{i,t}=\frac{MEI_{n-i,t}}{n-1}$$ (4)

#### Control variables

Other variables are defined as follows: GGI is used to measure the internal governance level of the company. In line with the previous literature [3, 22], this paper also examines the following variables that may affect stock price crash risk: annual standard deviation of weekly income (SIGMA), book-to-market (BM) value, total return on assets (ROA), opacity of corporate financial information (ACCM), that is, accrued profit manipulation by companies (which can be calculated by using the modified Jones model), the proportion of state-owned shares (SOE), the value of social contributions (SOCIAL), and the Fangsang Index (NERI) reflecting the market openness of the various provinces in China. Finally, this paper includes an annual dummy variable (YEAR) and an industry dummy variable (INDUSTRY) to control for annual and industry fixed effects, respectively.

### Piecewise regression models

A disadvantage of the traditional piecewise regression model is that it cannot determine whether the linear relationship between MEIs and stock price collapse risk is continuous. If there is no continuous linear relationship, the turning point cannot be determined. A new piecewise regression model designed by Shan et al. [43] solves this problem well. The MEI range is divided into three subsets according to percentage: less than 10% (MEI_L), 10% to 30% (MEI_M), and greater than 30% (MEI_H). To test Hypothesis 1, model 1 is set to test the relationship between MEIs and the risk of a stock price crash. The model is set as follows:

$$\begin{array}{l} NCSKEW=\alpha +\beta_{1}MEI(MEI\_L/MEI\_M/MEI\_H)+\gamma CONTROL\\ +TimeFE+IndustryFE+\varepsilon \end{array}$$ (5)

$$\begin{array}{l} DUVOL=\alpha +\beta_{1}MEI(MEI\_L/MEI\_M/MEI\_H)+\gamma CONTROL\\ +TimeFE+IndustryFE+\varepsilon \end{array}$$ (6)

**Fig 1:**
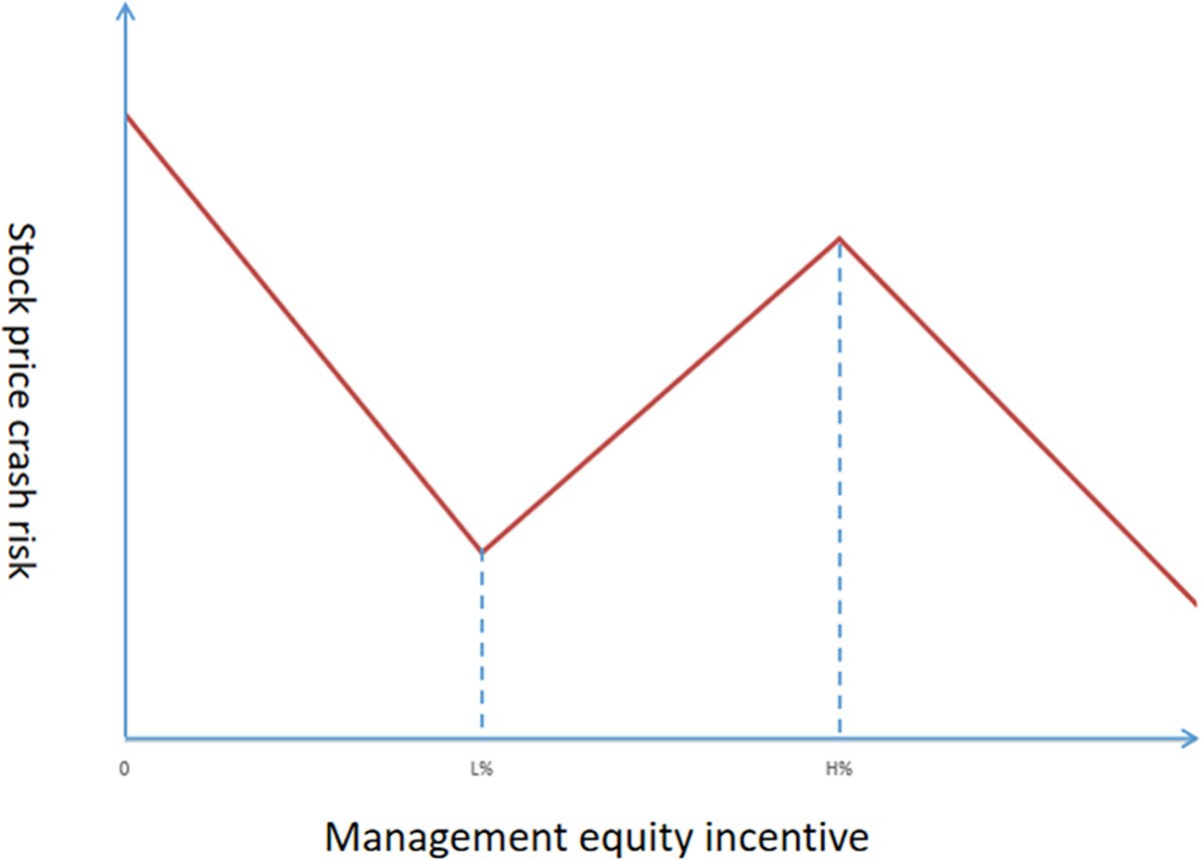
Piecewise regression model.

At this point, a figure with broken lines featuring both uptrends and downtrends (shown in Fig 1) can be obtained. The relationship between MEIs and the stock price crash risk in the L%-H% range trends in the opposite direction of the relationship in the other regions, and this complex relationship provides an explanation for a puzzle that had bewildered previous researchers: why do MEIs both increase and decrease stock price crash risk? Therefore, it is of great importance to determine L% and H%, that is, the upper and lower bounds of the gold watch region.

This result is similar to that of research on the relationship between Tobin’s Q and management ownership conducted by Morck et al. [7]. In Fig 1, the relationship between management ownership and stock price crash risk is negative in the 0%-L% range. This is because the interests of management and shareholders are consistent in this range. The harmonious relationship between the two sides reduces agency costs. Management is more likely to adopt a transparent management approach, which reduces the risk of a stock price crash. However, in the area from L% to H%, management equity incentives have the opposite effect. The expansion of management ownership arouses the vigilance and dissatisfaction of other shareholders and management also has a greater incentive to compete with shareholders for control of the company. In this region, there are fierce conflicts between management and shareholders, which increases agency costs. Management may adopt dishonest and opportunistic practices, thus raising the risk of stock price crashes. When management holds more than H%, it has basically gained control of the company, and the interests of shareholders and management tend to be consistent. Management may again adopt a transparent management approach and restrain opportunistic behaviour. The risk of a stock price crash decreases again.

### Mediating effects model

Based on the new three-step mediating effects model designed by Shan et al. [44], this paper further explores the impact of peer effects on the relationship between MEI and stock price crash risk, that is, Hypothesis 3. Previous studies have shown that the industry in which companies operate has an impact on management decision-making and stock price crashes, so this paper adds the variable PMEI (peer management equity incentive) to measure industry peer effects as a mediating variable in the regression model. The model is set as follows:

$$NCSKEW(DUVOL)=\beta_{0}+\beta_{1}MEI+\gamma CONTROL+TimeFE+IndustryFE+\varepsilon$$ (7)

$$PMEI=\alpha_{0}+\alpha_{1}MEI+\gamma CONTROL+TimeFE+IndustryFE+\varepsilon$$ (8)

$$NCSKEW(DUVOL)=\beta_{0}^{'}+\beta_{1}^{'}Residual_{MEI}+\beta_{2}PMEI+TimeFE+IndustryFE+\varepsilon$$ (9)

**Fig 2:**
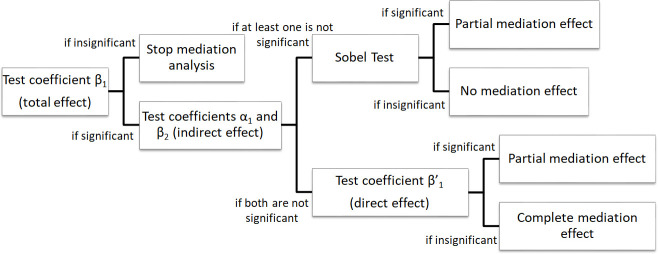
Mediating effects test procedure (Source: Wu et al. [44]).

The mediation test process is shown in Fig 2. First, the total effect of MEIs on stock price crash risk (β_1_, β_2_, β_3_) is measured, and further measurements are taken when β_1_ is statistically significant. Second, the indirect effect of MEIs (α_1_, β_2_) on the stock price crash risk is measured through peer MEIs. If the indirect effect (α_1_, β_2_) is significant, the direct impact of MEIs on the stock price crash risk (β_1_) is also significant. This shows that the effect of MEIs on the risk of a stock price crash is significantly reduced with the addition of peer MEIs, but the effect is not reduced to zero, so partial mediation has taken place. Another possibility is that there are significant indirect effects (any one of α_1_, α_2_, α_3_ and β_2_) and very weak direct effects (β_1_) between MEIs and the stock price crash risk, which means that a complete mediation effect exists. If at least one of the coefficients α_1_ or β_2_ is not statistically significant, the Sobel test is further conducted, and the indirect effect (α_1_ x β_2_) is assumed to be equal to zero (equivalent to β_1_−β^’^_1_). The critical ratio (S_α1β2_)^2^ that is generated by dividing (α_1_ x β_2_) by the standard error of the indirect effect is compared with the critical value of the standard normal distribution. If the Sobel test rejects the null hypothesis, it indicates that there is a partial mediation effect. The advantage of this model compared with the traditional three-step mediating effects model is that it adds the step of conducting a Sobel test, which can increase the model’s accuracy in identifying partial mediation effects.

## Empirical results and discussion

### Descriptive statistics

Table 1 shows the descriptive statistics for each variable. Of these statistics, the minimum and maximum values of NCSKEW are -2.358 and 1.262, respectively, and the average value is -0.337. The minimum and maximum values of DUVOL are -1.354 and 0.861, respectively, and the average value is -0.229, which is basically consistent with existing research on the Chinese stock market. However, there is a large gap between the stock markets on China and that on developing countries, which makes our research conclusions for the Chinese stock market distinctive to a certain extent. The average value of the MEI is 0.0455, and the maximum value is 0.615, which shows that MEIs in China are generally low, which is due to the special institutional background and social system in China. The mean and standard deviation of the PMEI and MEI variables are relatively close, which is in line with expectations.

**Table 1:** Descriptive statistics.

	(1)	(2)	(3)	(4)	(5)
Variable	N	Mean	SD	Min	Max
NCSKEW	6,499	-0.337	0.615	-2.358	1.262
DUVOL	6,496	-0.229	0.432	-1.354	0.861
MEI	6,562	0.0455	0.120	0	0.615
ROA	6,500	0.0418	0.0418	-0.104	0.192
ACCM	6,202	0.140	0.105	0.0085	0.768
MB	6,502	1.597	1.256	0.169	7.395
SIGMA	6,502	0.0609	0.0219	0.0240	0.131
SOE	6,565	0.0765	0.161	0	0.700
SOCIAL	6,575	0.118	0.500	0	4.37
NERI	6,515	7.382	1.822	2.64	10.923
PMEI	6,623	0.0617	0.0398	0.0000171	0.168

To reduce the bias in the estimated coefficients for the model, the following steps are performed. First, the continuous variables in the study are winsorized at the 1st and 99th percentiles. Second, the Pearson and Spearman rank correlations are used to test for the threat of multicollinearity. The pairwise correlation between the independent variables and the control variables is far less than 0.6 (not reported in the paper). The VIF coefficient is also calculated, with the maximum value being 1.67, which is lower than the critical value of 10. Therefore, the variables in this study are not threatened by multicollinearity.

### Regression analysis of Hypothesis 1

Hypothesis 1a predicts that there is a negative correlation between MEIs and stock price crash risk in the MEI golden handcuff regions, that is, MEI_L (0 < MEI < 10%) and MEI_H (30% < MEI < 100%). As shown in column (1) and column (3) of Table 2, the coefficients on both MEI_L and MEI_H are significantly negative (*β*_*1*_ = -1.504, p < 0.01; *β*_*3*_ = -0.758, p < 0.10). In column (2), the coefficient on MEI_M is significantly positive (*β*_*2*_ = 0.686, p < 0.10). Similarly, in column (3) and column (5), the coefficients on MEI_L and MEI_H are significantly negative (*β*_*1*_ = -0.826, p < 0.05; *β*_*3*_ = -0.480, p < 0.10). Hypothesis 1b predicts that there is a positive correlation between MEI_M (10%< MEI < 30%) and the stock price crash risk in the gold watch region. The coefficient on MEI_M is significantly positive (*β*_*2*_ = 0.422, p < 0.10). When the explained variable is changed, the relationship between MEIs and stock price crash risk still exhibits a trend of first falling, then rising, and then falling. In the low equity incentive golden handcuff regions, an increase in MEIs suppresses the stock price crash risk. As the MEI reaches its first turning point of 10%, it begins to enter the gold watch region, where the increase in the MEI increases the stock price crash risk. With the management entrenchment region ending at the turning point of 30%, the MEI has entered into a high equity incentive golden handcuff region, and the MEI once again suppresses the stock price crash risk. In the low-MEI golden handcuff region (0–10%) and the high-MEI golden handcuff region (30%-100%), management shareholding supports and coordinates the interests of managers and shareholders and reduces opportunistic management behaviour. In the gold watch region (10%-30%), management shareholding may encourage managers to conceal bad news and fraud. Therefore, both Hypothesis 1a and Hypothesis 1b are supported. In Table 2, the coefficients on MB are all significantly positive, and book-to-market value and stock price collapse risk are positively correlated, which is in line with our expectations. The coefficients on SIGMA are all significantly negative, indicating that SIGMA is negatively correlated with the risk of a stock price collapse, which is consistent with the findings in existing research. The coefficients on NERI are all significantly negative, meaning that NERI is negatively correlated with the risk of a stock price collapse. The higher the degree of economic openness where the listed company is located, the lower the risk of a stock price collapse is. The coefficients on BIG4 are significantly negative, and companies audited by the Big Four accounting firms have low stock price crash risk. Because the Big Four accounting firms have better reputations, they are more likely to detect and prevent fraud by the company’s management. The coefficients on SOE, ACCM and ROA are not significant.

**Table 2:** Regression results for Hypothesis 1.

	(1)	(2)	(3)	(4)	(5)	(6)
	NCSKEW			DUVOL		
	MEI_l	MEI_M	MEI_h	MEI_l	MEI_M	MEI_h
	0%-10%	10%-30%	30%-100%	0%-10%	10%-30%	30%-100%
MEI	-1.504***	0.686*	-0.758*	-0.826**	0.422*	-0.480*
	(-3.38)	(1.93)	(-1.93)	(-2.52)	(1.70)	(-1.77)
ROA	-0.00340	-0.00340	-0.00340	-0.120	-0.120	-0.120
	(-0.01)	(-0.01)	(-0.01)	(-0.70)	(-0.70)	(-0.70)
ACCM	0.00674	0.00674	0.00674	0.0192	0.0192	0.0192
	(0.08)	(0.08)	(0.08)	(0.31)	(0.31)	(0.31)
MB	0.0446***	0.0446***	0.0446***	0.0276***	0.0276***	0.0276***
	(4.82)	(4.82)	(4.82)	(4.11)	(4.11)	(4.11)
SIGMA	-7.095***	-7.095***	-7.095***	-4.864***	-4.864***	-4.864***
	(-11.32)	(-11.32)	(-11.32)	(-10.98)	(-10.98)	(-10.98)
SOE	0.0354	0.0354	0.0354	-0.0161	-0.0161	-0.0161
	(0.64)	(0.64)	(0.64)	(-0.40)	(-0.40)	(-0.40)
SOCIAL	-0.0118	-0.0118	-0.0118	-0.00464	-0.00464	-0.00464
	(-0.57)	(-0.57)	(-0.57)	(-0.33)	(-0.33)	(-0.33)
BIG4	-0.111***	-0.111***	-0.111***	-0.0839***	-0.0839***	-0.0839***
	(-3.85)	(-3.85)	(-3.85)	(-3.95)	(-3.95)	(-3.95)
NERI	-0.0155***	-0.0155***	-0.0155***	-0.0116***	-0.0116***	-0.0116***
	(-3.11)	(-3.11)	(-3.11)	(-3.26)	(-3.26)	(-3.26)
Year FE	YES	YES	YES	YES	YES	YES
Industry FE	YES	YES	YES	YES	YES	YES
Intercept	0.574***	0.574***	0.574***	0.365***	0.365***	0.365***
	(6.66)	(6.66)	(6.66)	(5.78)	(5.78)	(5.78)
*N*	4863	4863	4863	4870	4870	4870

*t* statistics in parentheses

* *p* < 0.1

** *p* < 0.05

*** *p* < 0.01

### Mediating effects analysis to test Hypothesis 2

There are asymmetries in information and responsibility between agents and principals: principals have no operational control over a company but have to bear the company’s profits and losses; agents have operational control over a company but do not bear the company’s profits and losses. This may cause agents to fail to devote themselves to collecting sufficient information and making correct corporate decisions. To protect their own interests, principals may urge agents to refer to the corporate decisions of other companies in the same industry. Listed companies are more willing to imitate group enterprises with good historical performance and large assets. According to social psychology research, imitation behaviour obeys three laws: the law of decline, the law of geometric series and the law of “first in, last out”. According to the law of decline, an inferior is more likely to imitate a superior. Large-scale leading enterprises can be considered superior due to their vast resources, their strong market position, and their dominant capacity. The policies of high-quality enterprises serve as a weathervane in the market, which makes them more likely to be imitated. On the other hand, small and medium-sized enterprises can be considered inferior because they have a weaker market position and have little influence on the market. Due to a lack of information, they are more likely to imitate other enterprises. High-quality enterprises generate industry norms in the management of other companies in the same industry, so peer effects could restrain stock price crash risk in an industry. Table 3 shows the mediating effect of peer effects on the relationship between manager shareholding and stock price crash risk. Specifically, columns (1) and (4) show the overall impact of MEIs on stock price crash risk, columns (2) and (5) show the impact of MEIs on PMEIs, and columns (3) and (6) show the direct impact of MEIs on stock price crash risk after controlling for peer effects. According to the process shown in Fig 2, the peer effect can be concluded to partially mediate the impact of MEIs on stock price crash risk, as shown in the following table.

**Table 3:** Mediation effects tests.

	(1)	(2)	(3)	(4)	(5)	(6)
Variable	NCSKEW	PMEI	NCSKEW	DUVOL	PMEI	DUVOL
MEI	-0.241***	0.00283***		-0.122**	0.00283***	
	(-2.90)	(2.72)		(-2.13)	(2.72)	
	[*β*_1_]	[*α*_1_]		[*β*_1_]	[*α*_1_]	[$\beta_{1}^{\prime }$]
Residual_MEI_			83.85***			43.76**
			(2.84)			(2.14)
			[$\beta_{1}^{\prime }$]			[$\beta_{1}^{\prime }$]
PMEI			-83.68***			-43.81**
			(-2.83)			(-2.15)
			[*β*_2_]			[*β*_2_]
Controls	Yes	Yes	Yes	Yes	Yes	Yes
Industry fixed effect	Yes	Yes	Yes	Yes	Yes	Yes
Year fixed effect	Yes	Yes	Yes	Yes	Yes	Yes
Sobel test	*β*_1_ = -0.241***			*β*_1_ = -0.122**		
	*α*_1_ = 0.00283***, *β*_2_ = -83.68***			*α*_1_ = 0.00283***, *β*_2_ = -43.81**		
	$\beta_{1}^{\prime }$ = 83.85***			$\beta_{1}^{\prime }$ = 43.76**		
	No Sobel test is required			No Sobel test is required		
Mediation effect	Partial mediation effect			Partial mediation effect		
N	4744			4749		

*t*-statistics in parentheses.

* *p* < 0.1

** *p* < 0.05, and

*** *p* < 0.01.

### Further tests

In this study, the range of entrenchment effects is examined based on the results of previous research. As in previous studies in which piecewise regression was used to evaluate MEIs [7, 40], in this study, three ownership areas are defined: MEI_L, MEI_M, and MEI_H. As far as we know, there are currently no studies on the management entrenchment region in MEIs in China, so the trial and error method is used to determine the range of the entrenchment effect. Various sensitivity analyses are carried out in this study to ensure the robustness of the main results. First, using the trial-and-error method and based on regression model 1, the impact of management entrenchment (or gold watch) on stock price crash risk is examined by using some of the entrenchment regions discussed in previous studies. For example, Morck et al. [7] found that the entrenchment region in the manager’s shareholding ratio in a US sample was 5%-25%. Based on a UK sample, Short and Keasey [45] identified the entrenchment region as 12%-40%, while Lennox [40] tested a number of entrenchment regions, including regions of 15%-40%. Lin and Liu [46] concluded on the basis of a sample of Hong Kong listed companies that the entrenchment region in the Hong Kong stock market was 20%-50%. In addition to these regions, other entrenchment regions, such as 25%-50% and 5%-60%, are also tested in this paper. The purpose of regressing the hypothetical relationship between these regions is to find further evidence to support the robustness to the main results from the tests of Hypothesis 1 and Hypothesis 2. The range of the test results for the identified entrenchment regions and turning points is shown in Table 4. Specifically, the entrenchment regions that were identified through significant turning points in the stock markets of other countries and regions (5%-50%, 10%-50%, 15%-50%, 20%-50%) are used to further estimate the gold watch region for Chinese A-share firms by means of trial and error. The tests in this study confirm that the entrenchment region that best describes the Chinese A-share market is 10%-30%. From the results in Table 4, the regression results from using other gold watch regions are not as good as those from using 10%-30% because using other regions does not guarantee that the coefficients on MEI_L, MEI_M and MEI_H are significant when NCSKEW and DUVOL are used as explanatory variables.

**Table 4:** Regressions for further hypothesis testing.

Entrenchment		NCSKEW			DUVOL		
		MEI_L	MEI_M	MEI_H	MEI_L	MEI_M	MEI_H
		(1)	(2)	(3)	(4)	(5)	(6)
	Sign	β_1_<0	β_1_>0	β_1_<0	β_1_<0	β_1_>0	β_1_<0
5%–25%	β	-1.027***	0.568	-0.398	-0.598**	0.408	-0.293
	t	(2.86)	(-1.39)	(1.28)	(2.34)	(-1.45)	(1.36)
10%–40%		-1.430***	0.464**	-1.960***	-0.726**	0.235	-1.055**
		(3.48)	(-1.99)	(2.68)	(2.40)	(-1.41)	(1.98)
12%–40%		-1.184***	0.593**	0.00529**	-0.541**	0.269	-0.832*
		(3.38)	(-2.20)	(-1.99)	(2.07)	(-1.39)	(1.80)
15%–40%		-1.055***	0.704**	-2.179***	-0.467**	0.288	-1.083**
		(3.75)	(-2.46)	(2.92)	(2.21)	(-1.37)	(1.97)
5%–50%		-0.819***	0.233	-14.83***	-0.413**	0.118	0.00367*
		(2.93)	(-1.37)	(4.78)	(2.05)	(-1.01)	(-1.96)
10%–50%		-1.252***	0.247	-15.65***	-0.621**	0.110	-7.773***
		(3.21)	(-1.38)	(4.70)	(2.18)	(-0.88)	(2.63)
20%–50%		-0.620***	0.429	-16.60***	-0.256*	0.141	-7.849***
		(2.89)	(-1.63)	(4.74)	(1.67)	(-0.78)	(2.61)
22%–50%		-0.998***	0.639***	-15.03***	-0.437**	0.252	-7.424***
		(4.02)	(-2.98)	(5.02)	(2.31)	(-1.59)	(2.66)
25%–50%		-0.433**	0.430	-16.29***	-0.187	0.144	-7.745**
		(2.43)	(-1.28)	(4.49)	(1.51)	(-0.64)	(2.53)
5%–60%		-0.433**	0.430	-16.29***	-0.187	0.144	-7.745**
		(2.43)	(-1.28)	(4.49)	(1.51)	(-0.64)	(2.53)

*t*-statistics in parentheses.

* *p* < 0.1

** *p* < 0.05, and

*** *p* < 0.01.

### Robustness tests

To test the robustness of the research results, with reference to the existing literature, the average age of management members(AGE) is used as an instrumental variable to re-examine whether MEIs have a significant impact on stock price crash risk. The robustness test results show that the coefficient on MEI is still significantly negative, and the coefficients in columns (1) and (2) of Table 5 are -1.309 and -1.373, respectively, which are significant at the 1% level. This result is reasonable because managers’ shareholdings of Chinese listed companies are concentrated in the range of 0–10%, a range in which management shareholding suppresses stock price crash risk. However, due to the limitations of the instrumental variable method, it is not possible to judge whether the results of the three coefficients are still robust after dividing MEI into MEI_L, MEI_M and MEI__H, so in this study, the Heckman two-step method is used to solve this problem.

**Table 5:** Robustness tests: Two-stage least squares (2SLS) model and Heckman two-stage model.

	(1)	(2)	(3)	(4)
	NCSKEW	DUVOL	NCSKEW	DUVOL
MEI	-1.309***	-1.373***		
	(-3.39)	(-4.87)		
MEI_L			-6.173***	-3.700***
			(-3.77)	(-3.15)
MEI_M			0.879**	0.499*
			(2.29)	(1.82)
MEI_H			-0.846**	-0.502*
			(-2.05)	(-1.69)
ROA	-0.142	-0.298	-0.0620	-0.145
	(-0.56)	(-1.60)	(-0.19)	(-0.63)
ACCM	0.0381	0.0467	0.193*	0.109
	(0.44)	(0.73)	(1.76)	(1.39)
MB	0.0343***	0.0154**	0.0820***	0.0474***
	(3.36)	(2.04)	(4.22)	(3.41)
SIGMA	-7.294***	-5.093***	-9.595***	-6.695***
	(-11.57)	(-10.99)	(-3.97)	(-3.86)
SOE	0.113*	0.0768		
	(1.71)	(1.58)		
SOCIAL	0.00540	0.0159	0.0126	0.0152
	(0.25)	(0.98)	(0.41)	(0.70)
NERI	-0.0206***	-0.0186***	0.00326	0.00174
	(-3.78)	(-4.59)	(0.28)	(0.21)
BIG4	-0.103***	-0.0716***	-0.143***	-0.0928***
	(-3.46)	(-3.24)	(-3.80)	(-3.45)
YEAR	YES	YES		
INDUSTRY	YES	YES		
IMR			1.294***	0.795**
			(2.85)	(2.45)
INTERCEPT	0.617***	0.429***	-0.687***	-0.384**
	(6.61)	(6.19)	(-3.12)	(-2.44)
*N*	4881	4888	3387	3400

*t*-statistics in parentheses.

* *p* < 0.1

** *p* < 0.05, and

*** *p* < 0.01.

The impact of MEIs on the stock price crash risk may differ due to differences in property rights between state-owned and non-state-owned enterprises, and the inhibitory effect of the industrial environment on the risk of a stock price crash may also differ depending on whether the state owns any shares. Therefore, in the robustness test, enterprises are grouped according to the nature of their property rights. The Heckman two-step method is used to test whether there are endogeneity problems. The regression results show that the significance and direction of the MEI_L, MEI_M and MEI__H coefficients remain unchanged—their coefficients were negative (-), positive (+), and negative (-)—and the model maintains the trend of falling, then rising, and then falling, which is consistent with the main analysis in our study. The coefficient on the IMR is still significant at the 5% level. Therefore, the results in the study can be considered robust.

## Conclusions

Based on the existing literature, this study further delves into the field of MEIs and stock price crash risk. By establishing piecewise regression models, the different management equity incentive regions are analysed. Specifically, the 10%-30% range for MEIs is defined as a gold watch region. In contrast, less than 10% and more than 30% are defined as a low-MEI golden handcuff region and a high-MEI golden handcuff region, respectively. The study found that the relationship between MEIs and stock price crash risk is negative in the low-MEI golden handcuff region and the high-equity incentive golden handcuff region, while the relationship between MEIs and the stock price crash risk is positive in the gold watch region. These findings suggest that the trend in the relationship between the risk of a stock price crash and MEIs is a broken line that decreases first, then rises, and then decreases. Due to the short-sightedness and opacity of management, managers in the gold watch region are more likely to engage in opportunistic activities, thus increasing the stock price crash risk for the company. In contrast, managers in the proposed golden handcuff regions are more likely to work with shareholders to protect the interests of the company, thus reducing the stock price crash risk. This study is not only in accordance with theoretical expectations but also highlights the impact of changes in jurisdictional areas.

In response to the increasingly stronger calls from academia and practitioners, we further analyse the mediating effect of external environmental constraints represented by industry peer management equity incentives on the relationship between management equity incentives and stock price crash risk. Through an mediating effects analysis, industry peer effects are found to reinforce the behavioural constraints on enterprise management and then reduce the probability of managers engaging in speculative behaviour, which plays a crucial role in reducing stock price crash risk.

## Supporting information

S1 Data (RAR)
