# Expression of Concern: Management equity incentives and stock price crash risk: “Golden handcuffs” or “gold watch”

**DOI:** 10.1371/journal.pone.0294800

**Published:** 2023-11-16

**Authors:** The PLOS ONE Editors

After this article [1] was published, concerns were raised that aspects of [1] are similar to pre-existing but unpublished work by another research group, and were published without the appropriate attributions and permissions.

In addition, concerns were raised about some aspects of the methodology in [1]. Specifically, that the data in S1 Data indicate that the managerial ownership consists of positive numbers, but that the code multiplied by –1 for each region of managerial ownership when the regions were divided into three distinct groups.

In response to queries about the methodology, the corresponding author stated that the results and hypothesis development/theoretical framework in [1] are correct because the two explained variables have a negative skewness problem. The corresponding author also stated that the method [2] used to divide mos into mos_l, mos_m and mos_h in [1] addresses cases where the explained variable has positive skewness, and to ensure the skewness of the explanatory variable agrees with that of the explained variable, mos_l, mos_m and mos_h were multiplied by-1 when calculating intervals, but not for regression. The corresponding author further noted that mos is a static variable, so the variable mei was used in [1]; mei is close to equal to mos in numerical terms, but provides a preferable theoretical explanation of why mei_l, mei_m and mei_h are inverted. An expert Academic Editor reviewed the methodology and S1 Data file and stated that if multiplication by -1 was done when calculating intervals, the methodology in [1] is adequate.

The complainants’ institution investigated the concern that aspects of [1] overlap with pre-existing work, and were published without the appropriate attributions and permissions. The institution advised that they did not find evidence of misconduct and the works in question used different datasets, but the algorithms and framework used in [1] were developed at, and are property of, the complainants’ institution. The institution also noted that documentary records confirmed that development of algorithms used in [1] involved researchers whose contributions are not disclosed on the *PLOS ONE* article. This institution’s investigation did not include interviews or research record reviews for the authors of [1], who are affiliated with a different university (Chongqing University).

The corresponding author refuted the claims and stated that the work reported in [1] was not performed at the complainants’ institution and stemmed from ideas developed independent of the complainants.

PLOS considers that the methodology concerns are resolved. However, we were unable to obtain input from Chongqing University as needed to clarify the concerns about ownership, permissions, and similarities to pre-existing unpublished work. Therefore, the *PLOS ONE* editors issue this Expression of Concern.
